# Toward Sustainable Food and Packaging Choices: Consumer Perception of Quality and Sustainability of Pulses Packaged in Metal, Glass, and Plastic

**DOI:** 10.1111/1750-3841.70585

**Published:** 2025-10-09

**Authors:** Lauren Thomas, Nomzamo N. Dlamini, Karen Cichy, Jeffrey Swada, Emily J. Mayhew

**Affiliations:** ^1^ Department of Food Science and Human Nutrition Michigan State University East Lansing Michigan USA; ^2^ Department of Consumer and Food Sciences University of Pretoria Pretoria South Africa; ^3^ Department of Plant Soil and Microbial Sciences Michigan State University East Lansing Michigan USA; ^4^ Sugarbeet and Bean Research Unit USDA‐ARS East Lansing Michigan USA

## Abstract

**ABSTRACT:**

Dry beans and other pulses have many environmental, nutritional, and health benefits—yet are continuously undervalued by consumers. Barriers to pulse consumption extend to ready‐to‐eat (RTE) canned options, as they are viewed as a low‐quality food. Additionally, consumers currently have misperceptions related to food packaging sustainability, as glass sustainability is overestimated, while plastic sustainability is underestimated, despite the sustainability of food packaging being very multifaceted. This study aimed to understand the impact of packaging on the acceptance (overall, appearance, texture, flavor liking) of RTE yellow beans and chickpeas within metal can, glass jar, and plastic pouch packaging. Perceptions of the product quality, product convenience, trust in product, and perceived sustainability of each product and packaging were also assessed, along with how consumers’ sustainability attitudes and demographics influenced packaging attribute responses. Blind‐coded serving samples processed within metal cans performed the most consistently for participants (*n* = 109) across sensory modalities, while yellow beans within plastic pouches and chickpeas within glass jars were least preferred. Glass jars were rated to be the highest quality and most sustainable packaging material, while plastic pouches were rated to be lowest quality and least sustainable, with most participants selecting to purchase the glass jar over the metal can or plastic pouch. Age, diet type, and certain sustainability attitudes were significant (*p* < 0.05) predictors of packaging attribute ratings. Results from this study can contribute to innovation and improvements to RTE pulse products, while also expressing a need for increased consumer education pertaining to packaging sustainability.

**Practical Applications:**

This study provides a better understanding of consumer perceptions related to retort‐processed pulse products, food packaging materials, and food packaging sustainability. This will allow for possible innovation opportunities to increase the availability and acceptability of pulses, a sustainable and nutritious food, as well as combat misconceptions related to food packaging sustainability.

## 1 Introduction

Pulses are defined as leguminous crops harvested solely for their dry grain and include dry beans, chickpeas, and lentils (Havemeier et al. 2017). Pulses have various environmental, nutritional, and health benefits. As a crop, legumes enrich the environment through their nitrogen‐fixing abilities, allowing for less reliance on synthetic fertilizers and associated carbon emissions (Castro‐Guerrero et al. 2016). They produce overall lower greenhouse gas emissions and water footprints than animal‐based protein sources (Semba et al. 2021) and can tolerate diverse environmental and soil conditions (Iriti and Varoni 2017). Pulses are also nutritionally dense in both macronutrients and micronutrients, as they are naturally high in proteins, carbohydrates, and fiber, naturally low in fat and sodium, and high in potassium, copper, phosphorus, manganese, iron, magnesium, and B vitamins (Mullins and Arjmandi 2021). Positive health benefits associated with frequent pulse consumption include reduction and prevention of obesity, diabetes mellitus, cardiovascular disease, and increased gut health (Didinger and Thompson 2022).

Despite these benefits, pulses are continuously undervalued by consumers, particularly compared to animal‐based protein products (Semba et al. 2021). Barriers to pulse consumption include disliking of sensory properties, lack of knowledge for preparing dry pulses (Winham et al. 2020), and potential intestinal discomfort (Messina 2014). Canning via retort processing provides a solution to the inconvenience of preparing dried pulses at home by producing ready‐to‐eat (RTE) pulse products. Moreover, those who eat canned foods frequently have been shown to have an overall higher intake of nutrient‐dense foods and healthier eating habits (Winham et al. 2020). However, there are also negative perceptions of canned/retort‐processed bean products among consumers, including that canned beans are not healthy, do not taste good, and contain preservatives (Heer and Winham 2020). Food packaging can also contribute to a consumer's negative perception of pulses, such as the perception that canned pulses are a low‐quality food (Melendrez‐Ruiz et al. 2019). While people typically refer to canned foods as food packaged within metal cans, canned foods can be sold in cans, glass jars, or soft food packaging materials (Zheng et al. 2023). Given the overall negative connotation pulses and canned foods currently have, there are clearly improvements to be made and opportunities to increase consumer acceptance of canned pulse products.

In recent years, there has been growing interest from government agencies, food manufacturers, and consumers to increase the sustainability of packaging materials (Oloyede and Lignou 2021). Sustainability of packaging materials is complex and varies widely across packaging materials. For example, glass and steel have a higher global warming potential (GWP) compared to plastic when initially processed, but glass and steel also have higher recycling rates and abilities to be recycled compared to plastic (Otto et al. 2021). When considering consumer perceptions of packaging material sustainability, Dlamini et al. (2024) found that consumers often idealize the sustainability of glass, overestimating its sustainability, while the sustainability of plastic is often underestimated (Otto et al. 2021).

The objectives of this study were to (1) assess the impact of retort processing within different packaging materials on consumer liking of processed pulses; (2) evaluate consumer perceptions of packaging material quality attributes; and (3) evaluate the effect of consumer attitudes toward packaging material sustainability on their perceptions of pulses packaged in either metal, glass, or plastic. Insights from this study can guide future pulse product development to increase consumer acceptance of canned pulses, as well as potentially demonstrate a need for increased consumer education to combat generalization of packaging material sustainability.

## 2 Materials and Methods

### 2.1 Bean Processing Protocols

**FIGURE 1:**
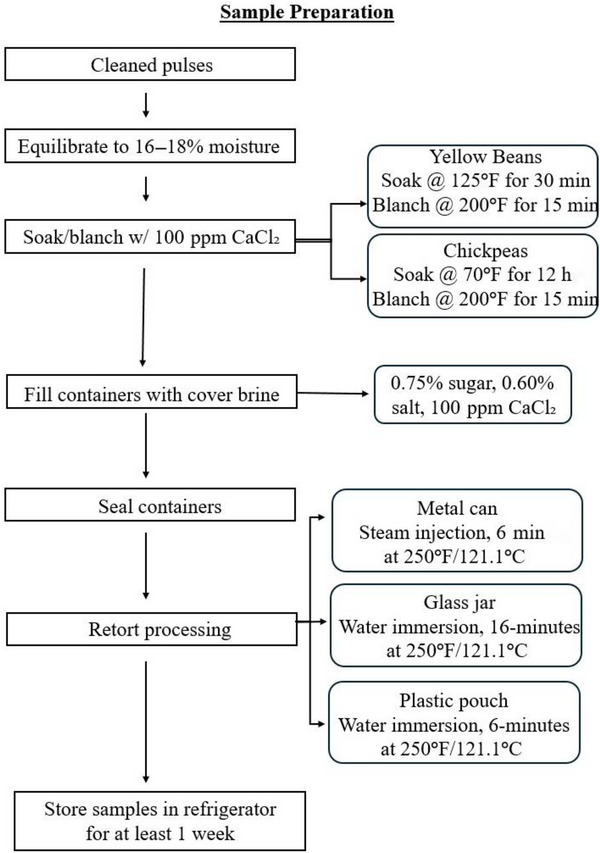
Flow chart for sample preparation and retort processing.

Kabuli chickpeas (‘Sierra’), harvested in September 2022 (Washington, USA), and yellow beans (RRY1803‐1‐1), harvested in September 2023 (Idaho, USA), were selected as the pulse cultivars to be retort processed. The rationale for selecting chickpeas is that they are one of the most frequently consumed legumes across the world, with consumption within the United States doubling between 2003 and 2018 (Rehm et al. 2023). Yellow beans are a minor market class of pulses within the United States, but their popularity has been increasing, and common bean breeders are becoming more interested in their potential for faster cooking times—contributing to increased global production potential (Wiesinger et al. 2018). The processing procedures were adapted and adjusted from Wang et al. (2022) and Wang et al. (2024), with procedures and sample preparation described in Figure 1. Cover brine used to fill each container consisted of 0.75% sucrose, 0.60% salt, and 100 ppm calcium chloride. Due to a processing error, salt was accidentally excluded from the brine used to fill glass jars for chickpea samples. Brine was heated to 200°F/93.3°C within a stainless‐steel steam kettle and allowed to cool to at least 120°F/48.8°C prior to filling and sealing containers. Upon filling and respective sealing of each container, samples were retort processed within a batch retort (ALLPAX 2402‐R3 Retort; Allpax, Covington, LA, USA) at the Michigan State University (East Lansing, MI, USA) Food Processing Complex (MSU FPC) at 250°F/121.1°C until a minimum thermal lethality of *F*_0_ = 6 min (Wang et al. 2024) was reached. Each bean variety and packaging material was processed separately, resulting in a total of six retort batches. After processing, samples were immediately placed into a walk‐in refrigerator (40°F/4.44°C) as an additional food safety measure, despite retort processing producing shelf‐stable food products (Jimenez et al. 2024). Within the refrigerator, samples were stored for at least 1 week prior to opening to better reflect the time a canned product might spend idle on a grocery store shelf prior to consumer purchasing.

#### 2.1.1 Sample Preparation

Retort‐processed bean samples were opened, decanted into a strainer, but not rinsed. To prepare samples to be given to study participants, 20–21 g of product was weighed into 2‐oz black sample cups with clear plastic lids, labeled with a three‐digit blinding code, and refrigerated the day before the study. Containers used for visual assessment were labeled with a product label and a three‐digit code. Generic product labels (Figure S1) were made for each bean variety, scaled to appropriately fit the front of each container. Each label contained the variety name, a photo of the processed bean, a small leaf graphic, a statement pertaining to the pulse's low carbon footprint (Drewnowski and Conrad 2024), and a standard weight of 250 g. Although samples varied in weight (Table 1), a standardized weight was used in an attempt to deter participants from favoring a packaging material based on its larger size or amount of product.

**TABLE 1:** Average weight of filled containers and emptied container contents for metal can, glass jar, and plastic pouch samples of retort processed yellow beans and chickpeas. Average weights were recorded from three samples of each variety/packaging material.

Variety	Packaging material	Unfilled weight (g)	Filled container weight (g)	Packaging percent of total weight
Yellow bean	Metal can	51.5	507.00	10.15%
	Plastic pouch	6.9	456.13	1.51%
	Glass jar	228.1	571.80	39.80%
Chickpea	Metal can	51.5	507.13	10.15%
	Plastic pouch	6.9	469.57	1.50%
	Glass jar	228.1	558.03	40.80%

### 2.2 Sensory Test Design

Regular pulse consumers from the greater Lansing, MI area were invited to participate in a 30‐min sensory evaluation session in November 2024 on the campus of Michigan State University (East Lansing, MI, USA). Eligible participants for the study were at least 18 years old, had no allergy to yellow beans, chickpeas, or wheat, and were regular consumers of beans. Interested participants completed a screening test through Sona Systems (Sona Systems, Bethesda, MD, USA), which required them to indicate that they were at least monthly consumers of beans, to successfully enroll. Participants were compensated with a $10 e‐gift card for participating in the study. The MSU Institutional Review Board (IRB) approved the sensory testing protocol (STUDY 00011439), and all participants gave informed consent prior to beginning the study.

**FIGURE 2:**
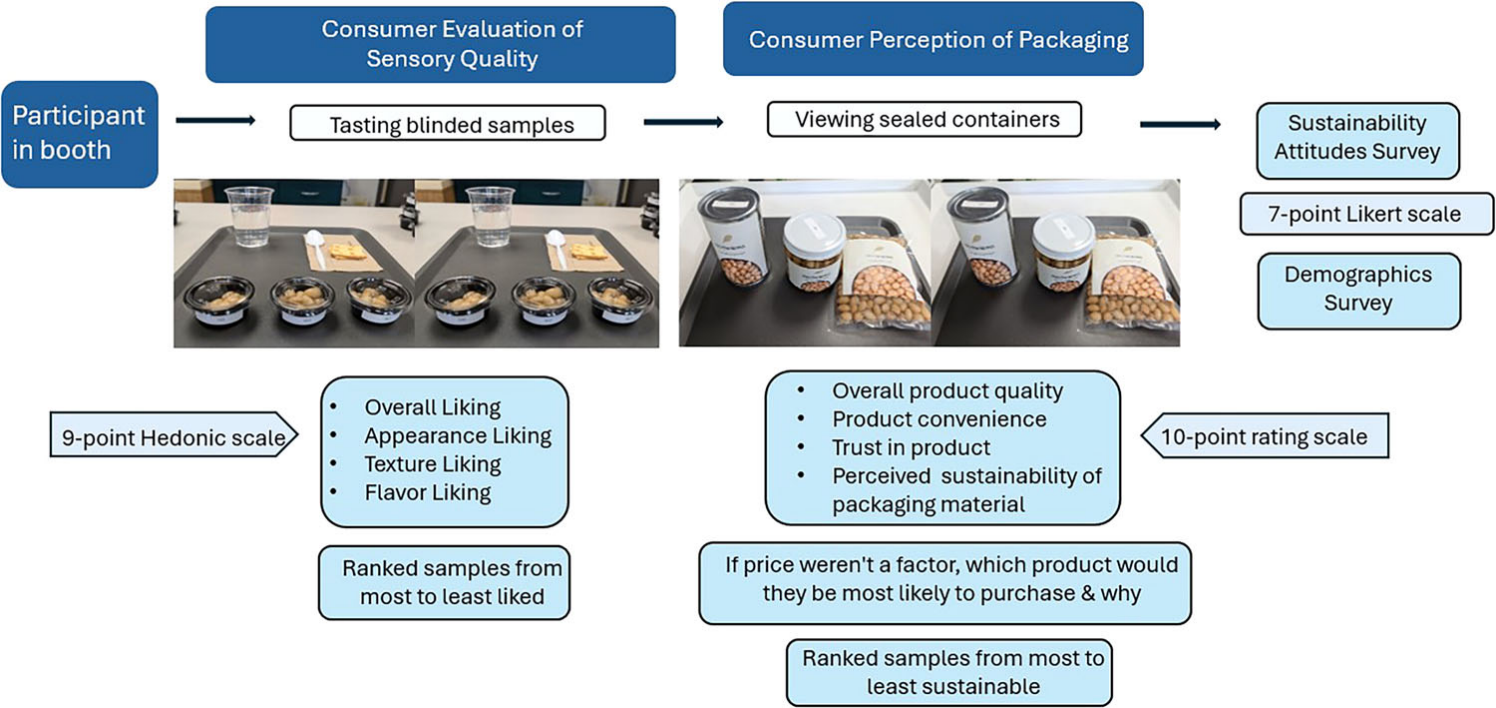
Overall sensory study design.

The design of the sensory study is shown in Figure 2. Each participant received a total of four trays: two trays (each with three samples) containing samples to taste, and two trays (each with three samples) containing samples for visual assessment, for a total of 12 samples. Participants were first presented with blinded pulse samples from a metal can, glass jar, and plastic pouch per bean variety, which were removed from the refrigerator and placed at room temperature for 30 min prior to serving. Participants were asked to rate their liking of the appearance, texture, flavor, and overall liking of the tasted samples on a scale from 1 (*Dislike extremely*) to 9 (*Like extremely*). In between each sample, participants were asked to cleanse their palates with room temperature water and unsalted saltine crackers, enforced by a 1‐min break period. Participants were then asked to rank all three samples from most to least liked. This process was repeated for the second tray of tasting samples.

In the next part of the session, participants received a tray containing an unopened metal can, a glass jar, and a plastic pouch of one pulse variety at a time. Participants were asked to visually assess and rate their perception of the overall product quality, product convenience, trust in the product, and perceived sustainability of the packaging material on a scale from zero to 10. Participants were then asked to select the sample they would most likely purchase if price was not a factor and briefly explain why through a prompted text response. Text response answers are valuable as they provide information, viewpoints, and thoughts in the participant's own words (Lindh et al. 2016). Participants concluded this section of the study by ranking all three samples based on their perception of most sustainable to least sustainable. This process was repeated for the second tray of unopened packages. The same seven sets of packaged samples were utilized throughout the entire day of the study.

Upon completion of tasting and visually assessing samples, participants completed a 22‐question survey related to their sustainability attitudes for packaging materials. Each question was answered on a scale from 1 (*Strongly disagree*) to 7 (*Strongly agree*), pertaining to a specific category (Table S1): gauging purchasing behaviors of products with environmentally friendly packaging, social influence, consumer perception of effectiveness, personal image concerns, quality of environmentally friendly packaging, and intention to buy products with environmentally friendly packaging, as published in Dlamini et al. (2024) to evaluate factors affecting sustainable consumerism. The purpose of these sustainability attitudes is to ultimately determine consumer intentions to buy products with environmentally friendly packaging by addressing these individual factors (Lan et al. 2023).

To conclude the study, participants answered demographic questions, including age, gender, a Check‐All‐That‐Apply (CATA) question for ethnic background, education level, employment status, diet type, a CATA question for what types of legumes and/or pulses they consume, a CATA question for how they consume/purchase pulses to consume, and how often they consume pulses. For the duration of the study, the design block and the order of questions were randomized across participants, with sample presentation order following a randomized complete block design—with blinded samples being tasted first, followed by packaging assessment, sustainability attitudes, and demographics last.

### 2.3 Statistical Analysis

Statistical analysis for this study was conducted using Statistical Package for Social Science (SPSS) (Version 30.0). Two‐way analysis of variance (ANOVA) (α = 0.05) was conducted on liking ratings for individual sensory modalities and packaging attributes separately for each bean variety. Post hoc pairwise comparisons were made using least significant difference (LSD), with significance beginning at *p* < 0.05. Additionally, a stepwise regression was conducted to use sustainability attitudes, participant age, gender, and diet type as predictors for participant ratings of product quality, product convenience, product trust, and perceived sustainability. Lastly, participant text responses regarding packaging preference were categorized into keywords to create word clouds with RStudio version R.4.4.1, using wordcloud2 package (0.2.1; Lang and Chein 2022) for each packaging type for each bean variety, visually displaying participant reasonings for preferred packaging based on the frequency of responses. Word clouds were created with a maximum of the top 12 responses for each variety and packaging type to ensure readability.

## 3 Results and Discussion

### 3.1 Subject Demographics

A total of 109 participants (*n* = 109) were successfully enrolled and completed the sensory test, with the average age being 32.6 (±16.8) years old. Participant demographic information is provided in Table S2. There was a higher proportion of females (61%) who completed the sensory test than males (38%). Participants who identified as White or Caucasian made up the largest ethnic background group (61%), followed by Asian or Pacific Islander (28%), Black or African American (7%), Hispanic or Latino (7%), and Native American (5%). Regarding education level, 100% of panelists had completed a high school education at minimum, with 91% of participants completing some form of secondary education after high school. While participants in this study were not asked about their income levels, those with higher education levels tend to have higher levels of income (Lindh et al. 2016).

Participants enrolled in this study were largely associated with following an omnivorous diet (72%), followed by flexitarian (9%), vegetarian (7%), other (6%), ovo‐vegetarian (2%), pescatarian (2%), and vegan (1%). Those who selected vegetarian, other, ovo‐vegetarian, and vegan diets will be described as “non‐meat eaters,” and those who identified as omnivores and pescatarians will be grouped as “meat eaters” for the analysis and discussion. Black beans were the most popular legume consumed (91%), followed by green beans (82%) and chickpeas (75%). Yellow beans were consumed by 38% of consumers. These findings are consistent with other studies, as Semba et al. (2021) found that although pulse consumption within the United States varies, black beans are consistently popular across all regions of the United States, and chickpeas are far more purchased by consumers than yellow beans. Within this study, most consumers indicated that they consume pulses one to three times per week (51%), while daily pulse consumption was rare (4%). Canned beans were the most common mode of consumption (86%). This is an expected finding, as canned food has become a core component of the American diet due to affordability, increased convenience, and comparable nutrient profiles to fresh food counterparts (Comerford 2015).

### 3.2 Sensory Test Results

#### 3.2.1 Overall Liking

**FIGURE 3:**
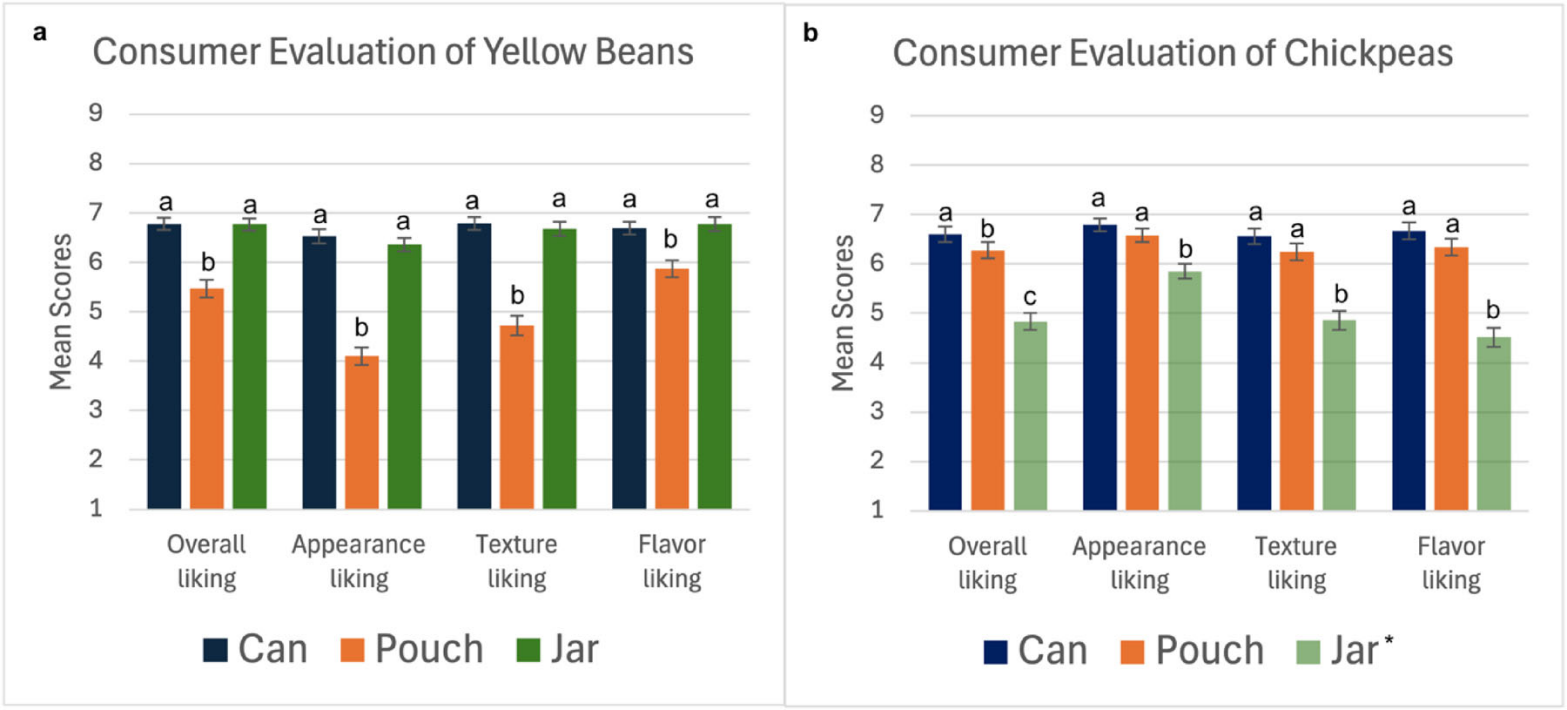
Mean consumer ratings for overall liking, appearance liking, texture liking, and flavor liking on a 9‐point Hedonic scale for yellow beans (a) and chickpeas (b) packaged in metal can (blue), plastic pouches (orange), and glass jars (green). Error bars represent the standard error of the mean (±1 SD). Groups not sharing the same letter are significantly different (*p* < 0.05). *Chickpea samples processed within glass jars (omitting salt) are shown with transparent coloring for visual differentiation.

Mean liking ratings by sensory modality for each pulse variety are shown in Figure 3 and Table 2. For yellow beans, participants gave the lowest ratings for samples processed within plastic pouches (*p* < 0.001) for every sensory modality, including overall liking. Overall liking ratings for samples within metal cans and glass jars did not differ significantly (*p* = 0.945). When participants were asked to rank the blinded samples from most liked to least liked, 47.7% of participants picked the metal can first, 39.4% the glass jar, and 12.8% the plastic pouch. The lower rating of yellow beans within plastic pouches is likely related to a noticeably softer texture, discussed below. With chickpeas, samples processed within glass jars were rated significantly lower (*p* < 0.001) across all sensory modalities, also including overall liking. Samples within metal cans were significantly (*p* = 0.046) more liked than samples within plastic pouches, followed by samples within glass jars. When asked to rank the blinded samples, 57% of participants picked the metal can sample as the most liked, 36.7% picked the plastic pouch sample, and 5.5% picked the glass jar sample. The omission of salt from the chickpea glass jar brine likely contributed to the overall low liking results, also discussed below. When considering overall liking of pulses to improve pulse consumption, Whittall et al. (2024) found that enjoyment or lack of enjoyment from eating pulses is linked to sensory properties, specifically taste and texture.

**TABLE 2:** Mean consumer ratings for overall liking, appearance liking, texture liking, and flavor liking on a 9‐point Hedonic scale. Groups not sharing the same letter are significantly different (*p* < 0.05).

Variety	Sensory modality				
Yellow beans	Packaging material	Overall liking	Appearance liking	Texture liking	Flavor liking
	Metal can	6.78^a^	6.53^a^	6.78^a^	6.68^a^
	Plastic pouch	5.46^b^	4.10^b^	4.72^b^	5.87^b^
	Glass jar	6.77^a^	6.35^a^	6.67^a^	6.77^a^
Chickpeas	Packaging material	Overall liking	Appearance liking	Texture liking	Flavor liking
	Metal can	6.61^a^	6.78^a^	6.56^a^	6.67^a^
	Plastic pouch	6.27^b^	6.57^a^	6.24^a^	6.33^a^
	Glass jar	4.83^c^	5.85^b^	4.86^b^	4.51^b^

#### 3.2.2 Appearance Liking

When considering sensory modalities individually, participant ratings for overall appearance liking showed similar trends to overall liking, where the sample with the lowest rating for appearance liking was also rated lowest for overall liking for both the yellow bean (plastic pouch) and chickpea (glass jar) samples. Byarugaba et al. (2020) found that when testing consumer acceptance and preferences of bean‐based sauces, appearance was a major driving factor compared to other sensory attributes, suggesting that product developers must pay attention to the appearance of their products to increase acceptance. It is particularly important to consider the appearance of pulse products when using transparent glass or plastic packaging, as transparent packaging allows consumers to evaluate the appearance of the product before making a purchase decision (Simmonds et al. 2018). Traditional metal can food packaging does not allow consumers to see the cooked samples until the can is opened. When further considering factors that determine appearance liking, Wang et al. (2022) assessed the appearance of retort‐processed pulse samples on a scale of 1 (*Unacceptable appearance, severe splits*) to 5 (*Excellent appearance, very few seed splits*) and found that samples with higher appearance scores had firmer textures and therefore less seedcoat splits. Given this finding, within this study, it is also likely that samples that were rated lowest for appearance liking also had more seedcoat splits and less firm texture. While no instrumental texture analysis was conducted, it was noted by researchers that the yellow beans that were retort processed within the plastic pouches, receiving the lowest ratings for overall appearance liking and texture liking, had noticeable seedcoat splits.

#### 3.2.3 Texture Liking

Participant ratings of texture liking were also similar to trends for the overall least liked samples. Uebersax and Hosfield (1985) previously determined ideal ranges of instrumental texture measurements for common bean market classes to be around 40–80 kilograms of force (kgf). These texture ranges are a useful reference tool for industry purposes, as beans below the 40 kgf measurement are likely too soft and would be considered overcooked by consumers, while beans above the 80 kgf measurement are likely too firm and would be considered undercooked by consumers. A companion study (Thomas et al. 2025) conducted instrumental texture analysis of the same varieties and seed sources used within this study. Samples with the lowest texture ratings were likely considered too soft by participants, and this soft texture also resulted in lower appearance liking scores due to increased numbers of seedcoat splits. Given that the chickpea samples processed within glass jars without salt were rated significantly lower in both appearance liking and texture liking, it is likely that the lack of salt impacted these attributes. Pedone et al. (2024) previously found variations in the hardness and visual appearance of beans soaked before cooking within varying concentrations of salt, with results being cultivar and concentration dependent. Despite these attributes not being directly measured using instrumental analysis within this study, it is possible that the lack of salt within these samples contributed to reduced mean scores of other attributes besides overall liking when compared to samples processed with salt. Additionally, variations in retort processing times and cooking within different packaging materials have previously been shown to impact product textures. Upon the same retort processing times and temperature conditions using metal cans, glass jars, and plastic pouches, yellow beans have shown to have softer texture measurements compared to chickpeas (Thomas et al. 2025). Because both pulse cultivars in this study were again subjected to the same processing times and temperatures for each given packaging material, it is likely that a softer texture was generally observed within yellow bean samples compared to chickpea samples. Wang et al. (2025) previously found that yellow beans tended to be firmer in texture when processed for shorter amounts of time within plastic pouches compared to metal cans. In this study, the processing time of samples to reach a minimum target thermal lethality of *F*_0_ = 6 min (Wang et al. 2024) for plastic pouches was shorter (6 min) than glass jar samples (16 min) but the same as the metal can samples (6 min). The reduced processing time requirements in this study are likely attributed to the small‐scale retort within MSU FPC that was used for the duration of the study, and the comparable processing time between the metal can and plastic pouch is likely due to the thin profile of the plastic pouches, allowing for rapid heating (Jung et al. 2023). The thin plastic pouches also lack structural rigidity, offering less protection to the product during processing and postprocessing handling, likely resulting in increased seedcoat splits due to product damage prior to consumption, as more seedcoat splits result in decreased firmness (Uebersax and Hosfield 1985). Retort pouches are gaining commercial acceptance due to their storage and preparation efficiency, as well as savings in packaging cost, transportation, and energy (Pal et al. 2019). RTE pulse manufacturers interested in utilizing retort plastic pouches should further explore processing methods to ensure consistently high‐quality products that are free of damage. Across all packaging materials, manufacturers can also consider retort processing for shorter amounts of time to improve canning quality attributes, such as texture (Bassett et al. 2020), while still carefully ensuring product safety. Further optimization of cooking times for specific pulse varieties in their respective packaging material would likely result in improved sensory qualities, particularly on a commercial scale. In this study, the standardized retort processing times utilized for each packaging material may not have been optimal for both cultivars. However, the observed overcooking of yellow beans within plastic pouches is still representative of the product quality risks of utilizing less protective and transparent packaging materials, where product defects are more pronounced.

#### 3.2.4 Flavor Liking

Lastly, flavor liking also followed similar trends to the other sensory modalities. It was expected that chickpeas processed in glass jars would have the least liked flavor, due to processing error leading to the lack of flavor‐contributing ingredients, but the significantly lower (*p* < 0.001) participant ratings of the salt‐free product are still a useful finding. Liem et al. (2011) previously predicted that sodium reduction will decrease consumer preference for foods, and when considering sodium reduction in foods for health purposes, the food industry uses sodium to “impart flavor” to food products. This finding was true within this study, as participants preferred samples that contained salt, contributing to their flavor liking as well as overall liking. For yellow beans, given the visible damage to yellow beans processed within plastic pouches and low appearance and texture scores, it is possible that a halo effect resulted in significantly lower flavor liking ratings or that the overcooking that negatively impacted appearance and texture also negatively impacted the flavor of the beans.

### 3.3 Packaging Quality Attributes

#### 3.3.1 Product Quality

**FIGURE 4:**
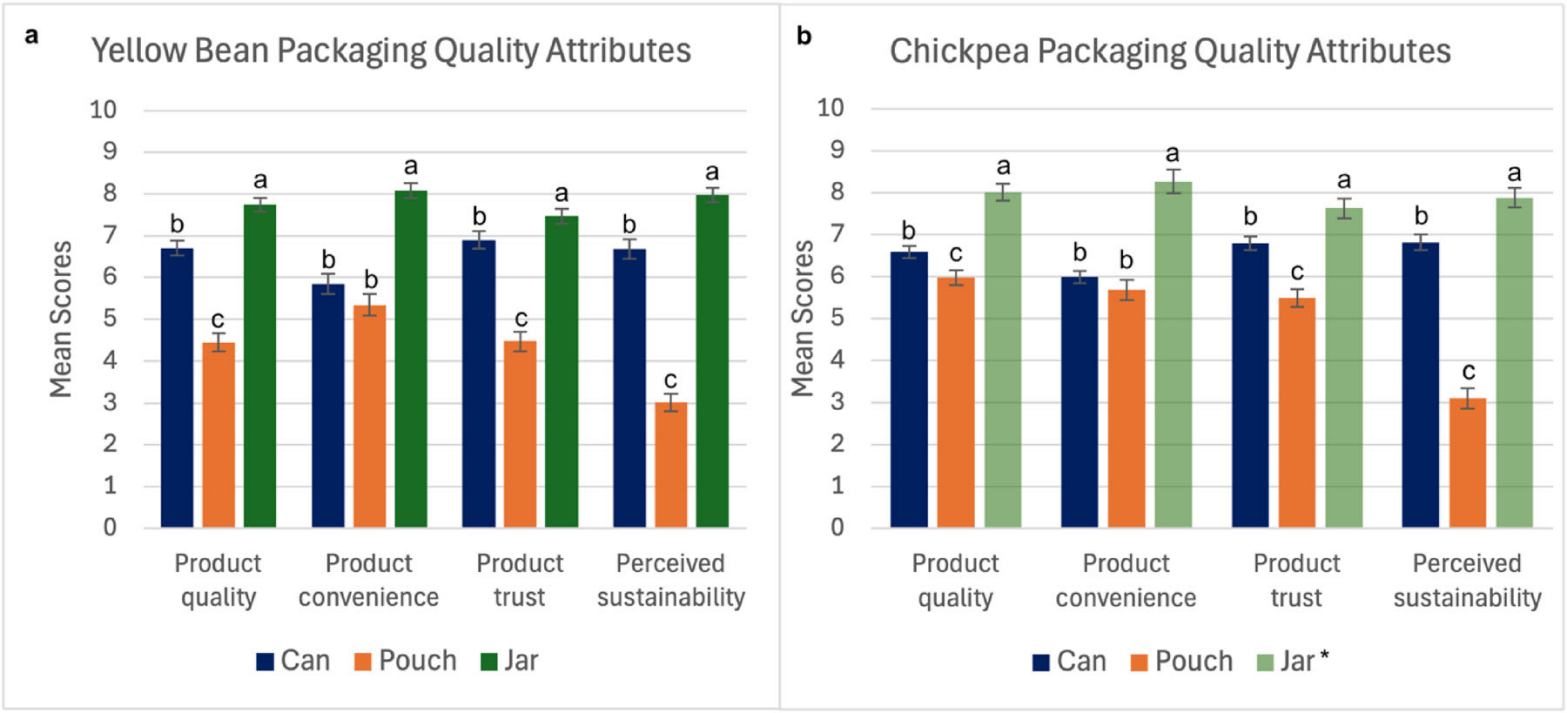
Mean consumer ratings for product quality, product convenience, trust in product, and perceived sustainability between packaging materials on a 10‐point rating scale for yellow beans (a) and chickpeas (b) packaged in metal can (blue), plastic pouches (orange), and glass jars (green). Error bars represent the standard error of the mean (±1 SD). Groups not sharing the same letter are significantly different (*p* < 0.05). *Chickpea samples processed within glass jars (omitting salt) are shown with transparent coloring for visual differentiation.

Packaging quality attribute ratings for unopened yellow bean and chickpea samples within all three packaging materials are shown in Figure 4 and Table 3. For both varieties, there were significant differences (*p* < 0.001) in perceived product quality for metal can, glass jar, and plastic pouch packaging, where participants rated the glass jar samples to have the highest product quality, followed by the metal can and then the plastic pouch. When analyzing consumer preferences of common packaging materials, Dlamini et al. (2024) also found that glass was the most preferred food packaging material for orange juice, while plastic and aluminum were the least preferred. Fernqvist et al. (2015) found that consumers viewed plastic packaging as a low‐quality packaging material for packaged potatoes. Lastly, Ruggeri et al. (2022) found that when evaluating consumer interest in purchasing wine in glass bottles or aluminum cans, 83% of respondents indicated that the aluminum packaging is a low‐quality material compared to glass, and 43% were concerned about the aesthetics of the aluminum packaging. Although from different food products, these studies show that consumers tend to view glass packaging as having overall higher quality, while metal and plastic packaging are often considered to be lower quality, as reflected by the results of this study.

**TABLE 3:** Mean consumer ratings for product quality, product convenience, product trust, and perceived sustainability on a scale from 0 to 10 where 10 is the best rating. Groups not sharing the same letter are significantly different (*p* < 0.05).

Variety	Packaging attribute				
Yellow beans	Packaging material	Product quality	Product convenience	Product trust	Perceived sustainability
	Metal can	6.70^b^	5.84^b^	6.89^b^	6.68^b^
	Plastic pouch	4.45^c^	5.33^b^	4.46^c^	3.00^c^
	Glass jar	7.74^a^	8.08^a^	7.46^a^	7.98^a^
Chickpeas	Packaging material	Product quality	Product convenience	Product trust	Perceived sustainability
	Metal can	6.58^b^	5.99^b^	6.79^b^	6.81^b^
	Plastic pouch	5.97^c^	5.68^b^	5.49^c^	3.10^c^
	Glass jar	8.01^a^	8.27^a^	7.63^a^	7.88^a^

#### 3.3.2 Product Convenience

When considering product convenience, consumers again favored glass jars over metal cans and plastic pouches. For both varieties, consumers rated glass jars to be most convenient, while metal cans and plastic pouches were viewed as less but equally convenient. This result was unexpected, as one advantage of retort pouches is their ability to be easily opened (Devadason et al. 2014), without the use of a can opener. Another possible consideration for product convenience is the size of each container, as the glass jar was the smallest in size (12 oz), while the plastic pouch was the largest (22 oz). Packaging size has previously been shown to be an indicator of convenience (Lindh et al. 2016), as the larger size packaging is bulkier and would take up more space within one's cabinet, while also containing a larger portion of food to be consumed than a smaller package (Hieke et al. 2016). The lid of the glass jar could also be used to easily reseal the container after opening, while the metal can and plastic pouch cannot be easily resealed within the original container once opened, thereby impacting convenience. Ensuring that plastic pouches are resealable may increase the chances of acceptance if such a product is to be commercialized.

#### 3.3.3 Trust in Product

When assessing overall trust in the food product in each packaging material, participants found glass jars to be more trustworthy than metal cans (*p* = 0.018) for yellow beans but found plastic pouches to be significantly less trustworthy (*p* < 0.001). For chickpeas, glass jars were viewed as more trustworthy (*p* = 0.001) than metal cans, and metal cans more trustworthy (*p* < 0.001) than plastic pouches. While food quality refers to tangible sensory characteristics of food, including taste, appearance, and texture, food safety is also considered an attribute of food quality, pertaining to the safe handling and storing of food, protecting the product from harmful biological, chemical, or physical contaminants (Wu et al. 2021). Overall perception of food product safety is subjective and varies by consumer, despite objective safety, which is assessed by scientists and experts (Grunert 2005). This was shown in this study, as all three products were processed to commercial sterility via retort processing and were equally safe to consume; yet, there was varying trust in the product despite no inherent safety differences. Consumers are becoming increasingly more aware of the harmful implications of plastic, including environmental and health implications, resulting in “plastic bashing” and an increased demand for nonplastic food packaging (Otto et al. 2021), possibly explaining the lowest perceived trust in plastic pouches. Similarly, the epoxy resin linings of metal cans often contain bisphenol A (BPA), and exposure through food packaging has been recognized as a primary source of BPA accumulation in humans (Manzoor et al. 2022). It is possible that BPA‐related health concerns contributed to reduced participant trust in metal cans within our study. Additionally, Isanovic et al. (2023) found that differences in sensory characteristics of a food allow consumers to believe that the food's nutrition, health, or quality has been compromised and that the first impression of a food may ultimately lead to mistrust. As previously discussed, the packaging product quality was ranked lowest for yellow beans and chickpeas packaged in plastic pouches, likely contributing to an initial lack of trust. It is an important consideration that the same packaging samples consumers received were used throughout the duration of the study, and the plastic pouches ultimately underwent the most “wear and tear” from being handled, resulting in increasingly more noticeable product deformation, likely also impacting perception of product trust and therefore product quality.

#### 3.3.4 Perceived Sustainability

Lastly, perceived sustainability of packaging varied across all three packaging materials. For both varieties, each packaging material was significantly different (*p* < 0.001) from one another, with glass jars rated as the most sustainable, followed by the metal cans, and the plastic pouches rated as the least sustainable. The idealization of glass has been observed in past studies, where Dlamini et al. (2024) also found that consumers perceived glass to be the most preferred and the most sustainable, while plastic and aluminum were the least preferred and viewed to be the least sustainable. Otto et al. (2021) found that consumers judge the sustainability of packaging materials based on natural‐looking packaging, design, theoretical recyclability, reusability, and biodegradability. As discussed previously, the sustainability of packaging material is complex. Production of packaging materials results in various GWP, with glass and metal production having overall higher GWP compared to plastic (Otto et al. 2021). For some energy expenditure considerations, Wang et al. (2024) noted that retort pouches are more energy efficient due to their thin profile, resulting in shorter processing times. However, within this study, metal cans and plastic pouches underwent the same retort processing times, while glass jars had the longest processing times. Reducing processing times could help in minimizing deterioration of product quality characteristics and in optimizing energy consumption (Pursito et al. 2020). When further considering energy expenditure, Dlamini et al. (2024) discussed that plastic is lighter in weight than glass, requiring less energy to transport compared to heavier glass products. In this study, the plastic pouch packaging contributed to approximately 1.5% of the total product weight, the metal cans approximately 10%, and the glass jars approximately 40% (Table 1). When considering recycling, in 2018, the Environmental Protection Agency found that the recycling rate was 70.9% for steel cans, 31.3% for glass jars, and 8.7% for plastic, noting that plastic recycling is complex, as only certain plastics can be recycled, according to the United States Environmental Protection Agency (2024). The epoxy lining within steel cans to prevent corrosion may impact its ability to be recycled, while glass has the ability to be continuously recycled (Otto et al. 2021). Furthermore, the lack of recycling and improper handling of plastic packaging largely contribute to land and water pollution (Ibrahim et al. 2022). Despite the complexity and lack of a straightforward answer to packaging sustainability, participants in this study viewed glass to be the most sustainable and plastic the least, following the results of Dlamini et al. (2024) and Otto et al. (2021). While identifying one material to be more sustainable than another is outside of the scope of this study, it is evident that there is misinformation among consumers regarding packaging sustainability, resulting in consumer convictions that are not supported by environmental impact data.

### 3.4 Sustainability Attitudes

#### 3.4.1 Product Quality

When assessing the predictivity of sustainability attitudes and demographic variables (Tables 4 and 5) on perceived product quality, there were no significant (*p* > 0.05) predictors for overall product quality for yellow beans. However, there was a positive association between the perception of the quality of chickpeas within plastic pouches and consumer age, indicating that older consumers rated chickpeas within plastic pouches to be of higher quality. There were no significant predictors associated with chickpeas packaged within metal cans or glass jars.

**TABLE 4:** Product quality, product convenience, product trust, and perceived sustainability of metal can, glass jar, and plastic pouch packaging materials for yellow beans predicted by sustainability attitudes, age, gender (man or woman), and diet type (meat eater vs. non‐meat eater): A stepwise regression (*n* = 108). Only statistically significant (*p* < 0.05) results are shown.

Standardized beta (*β*) values												
Packaging attribute	Container	Purchase behavior	Image concern	Packaging quality	Social influence	Effectiveness perception	Age	Gender	Diet type (non‐meat)	*R* ^2^	*p*‐value
Product quality	Metal can										
	Plastic pouch										
	Glass jar										
Product convenience	Metal can										
	Plastic pouch								−0.356	0.127	<0.001
	Glass jar						−0.200			0.040	0.038
Trust in product	Metal can										
	Plastic pouch				0.280				−0.302	0.124	<0.001
	Glass jar			0.229						0.052	0.017
Perceived sustainability of packaging material	Metal can								−0.202	0.041	0.036
	Plastic pouch			−3.83	0.302					0.136	<0.001
	Glass jar			0.269			−0.221			0.123	0.001

**TABLE 5:** Product quality, product convenience, product trust, and perceived sustainability of metal can, glass jar, and plastic pouch packaging materials for chickpeas predicted by sustainability attitudes, age, gender (man or woman), and diet type (meat eater vs. non‐meat eater): A stepwise regression (*n* = 108). Only statistically significant (*p* < 0.05) results are shown.

Standardized beta (*β*) values											
Packaging attribute	Container	Purchase behavior	Image concern	Packaging quality	Social influence	Effectiveness perception	Age	Gender	Diet type (non‐meat)	*R* ^2^	*p*‐value
Product quality	Metal can										
	Plastic pouch						0.310			0.096	0.001
	Glass jar										
Product convenience	Metal can										
	Plastic pouch								−0.231	0.053	0.016
	Glass jar										
Trust in product	Metal can								−0.197	0.039	0.041
	Plastic pouch						0.265		−0.231	0.132	<0.001
	Glass jar	0.274								0.075	0.004
Perceived sustainability of packaging material	Metal can						0.199			0.040	0.039
	Plastic pouch			−0.353	0.305		0.231			0.193	<0.001
	Glass jar					0.253				0.064	0.008

#### 3.4.2 Product Convenience

For both pulse varieties, non‐meat eaters found plastic pouches to be less convenient. Kim et al. (2022) found that convenience as a food choice motive is significantly more important to vegetarians than omnivores. Dlamini et al. (2024) also found that non‐omnivores valued plastic packaging less than omnivores. Furthermore, there was a negative association between the convenience of yellow beans packaged within glass jars and age, indicating that older consumers viewed glass jar packaging to be less convenient. Świda et al. (2018) found that the age of consumers affects their shopping habits and product choices, and 77.3% of older participants in their study indicated they preferred traditional and easy‐to‐open packaging. It is possible that older individuals associate glass jars with being less convenient due to the increased product weight associated with glass packaging, as well as glass jars often being difficult to initially open for consumers of all ages. When analyzing consumer perceptions of packaging, Lindh et al. (2016) found that key factors for consumer packaging choices include characteristics such as “easy to reseal,” “easy to open,” “packaging size,” and less about “product protection” and “product information”—although primarily convenience based, they can unintentionally contribute to increased sustainability when both are aligned, such as a resealable packaging container encouraging less food waste. In the context of this study, this information is useful, as participants showed a preference for functional, convenient, and sustainable packaging materials. With chickpeas, there were no additional predictors for package convenience across all packaging materials, assessed sustainability attitudes, or demographic characteristics.

#### 3.4.3 Trust in Product

When considering trust in product across all three packaging materials, the non‐meat eaters were less likely to trust the yellow beans within metal cans and chickpeas within plastic pouches. However, trust in chickpeas within plastic pouches again increased with age. Trust for chickpeas within glass jars was positively associated with environmentally friendly purchasing behaviors, indicating that people who believe purchasing environmentally friendly packaging benefits the environment are more likely to trust glass. Additionally, Oloyede and Lignou (2021) also found that consumers “will go for glass instead of plastic because I feel glass is more sustainable.” Participants with positive attitudes toward the quality of environmentally friendly packaging, meaning people who believe packaging materials can protect the appearance and quality of a product while also benefiting the environment (Lan et al. 2023), were more likely to trust yellow beans within glass jars. Participants whose sustainability behaviors are informed by social influence were more likely to trust and have a positive perception of the sustainability of yellow beans within plastic pouches. Lan et al. (2023) explained that the relationship between social influence and purchase decisions is related to the information one collects from their various social sources, and ultimately found that social image does contribute to purchasing behaviors, but social influence for environmentally friendly behaviors is only effective when one is aware of the effectiveness of sustainable products, resulting in positive attitudes.

#### 3.4.4 Perceived Sustainability

As the age of participants increased, perception of sustainability for chickpeas packaged within metal cans and plastic pouches increased. On the contrary, perceived sustainability of yellow beans within glass jars decreased with age, indicating that younger consumers may view glass jars as more sustainable and metal cans and plastic pouches as less sustainable. Those who value the quality of environmentally friendly packaging materials were less likely to have a positive perception of the sustainability of chickpeas and yellow beans in pouches. However, those whose sustainability attitudes were socially influenced were more likely to have a positive perception of the sustainability of chickpeas and yellow beans packaged in plastic pouches. These results align with packaging quality attributes; glass jar packaging was rated to be more sustainable than plastic pouches for both pulse varieties, which agrees with previous studies where consumers found glass jars to be both the highest in quality and most sustainable, and plastic and aluminum the lowest in quality and least sustainable (Dlamini et al. 2024). Chickpeas within glass jars were also associated with being an effective and sustainable packaging material, again affirming why glass jars were selected to be the most sustainable packaging material by the majority of participants in this study. Non‐meat eaters found yellow beans within metal cans to be less sustainable, and the perception of metal packaging being less sustainable than other packaging materials is likely again related to overestimation of glass sustainability (Lindh et al. 2016), despite non‐omnivores (vegetarian, lacto‐vegetarian, ovo‐vegetarian, and vegan) being significantly more likely to consume legumes and canned vegetables than omnivores (Jedut et al. 2023).

### 3.5 Consumer Reasons for Packaging Choice

Without considering price, participants were asked which packaging material they would be most likely to purchase for each pulse variety. With chickpeas, glass jars were the most preferred packaging material (61.5%), followed by metal cans (30.3%) and then plastic pouches (8.3%). Participant preferences closely followed those for yellow beans, where glass jars were again the most preferred (67%), followed by metal cans (31.2%) and then plastic pouches (1.8%). Plastic pouches were slightly more accepted for chickpeas compared to yellow beans, but glass jars were significantly more preferred across both varieties.

**FIGURE 5:**
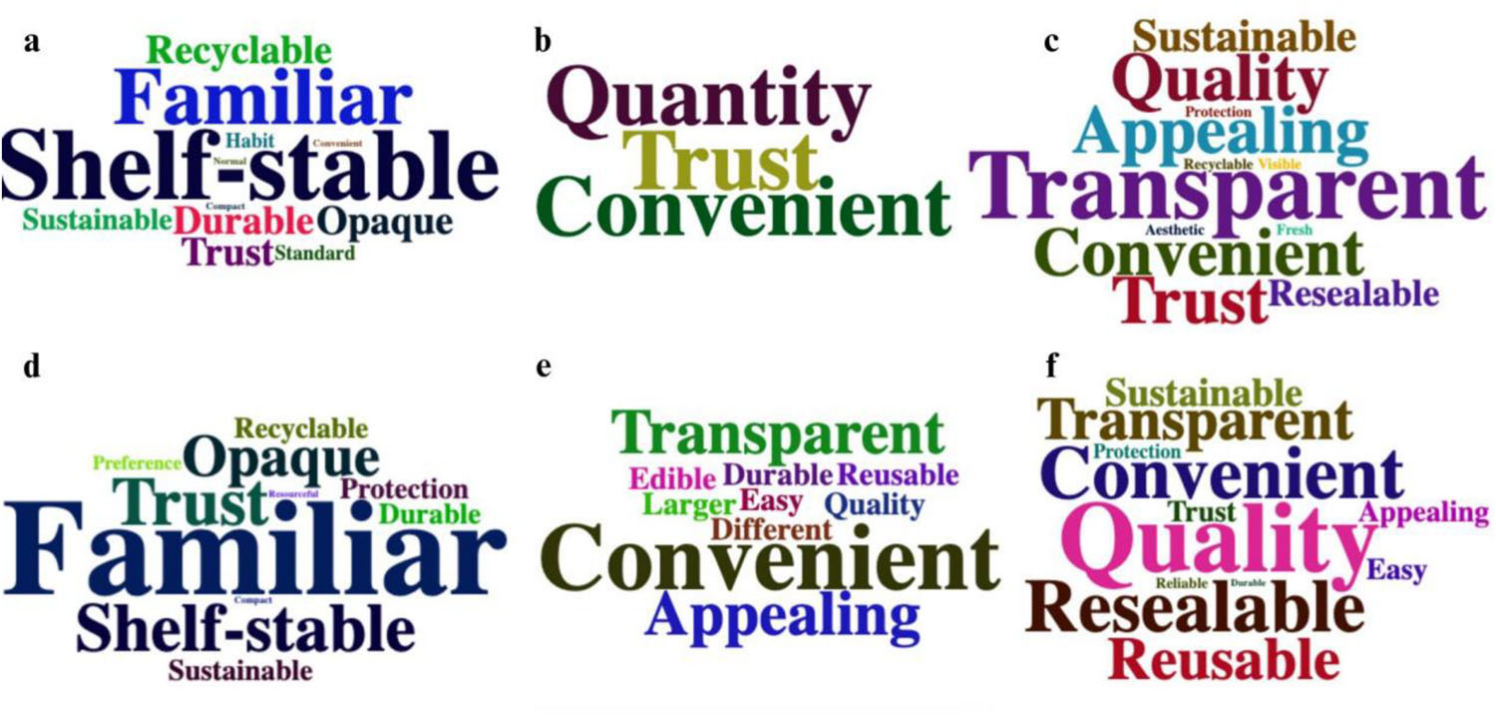
Word cloud explaining consumer reason for most preferred packaging type for yellow beans within metal cans (a, *n* = 34), plastic pouches (b, *n* = 2), and glass jars (c, *n* = 73) and chickpeas within metal cans (d, *n* = 33), plastic pouches (e, *n* = 9), and glass jars (f, *n* = 67). Terms with larger font were cited more frequently.

Participant text responses explaining their reasons for choosing each packaging material were generally similar between both bean varieties (Figure 5). Metal cans were associated with being familiar, shelf‐stable, durable, protective, and recyclable. It was also noted that some participants preferred the opaque packaging of metal cans compared to transparent glass jars or plastic pouches, as they felt it was unnecessary for the pulses to be packaged in transparent materials. Metal can food packaging is known for being very durable and protective, as it is a rigid material that provides a barrier against light, gas, and moisture (Deshwal and Panjagari 2020). Additionally, approximately 75% of bean products sold within the United States are sold within metal cans (Papanikolaou et al. 2024), highlighting the strong consumer familiarity with beans packaged within metal cans. Glass jars were associated with being high‐quality, transparent, reusable, resealable, and sustainable. It was evident that a major consideration for glass jar packaging material was the ability to reseal the lid of the jar or to repurpose the jar. Lindh et al. (2016) found that functional properties of packaging impact consumer purchasing decisions, and 27% of their survey respondents stated that the ability to reseal a package contributes to its everyday convenience. Pertaining to sustainability efforts, the 3R strategy is a waste management strategy—with an emphasis on reducing, reusing, and recycling—that consumers feel should be implemented to make food packaging more sustainable (Oloyede and Lignou 2021). Greenwood et al. (2021) found that consumers are more willing to reuse glass packaging compared to plastic packaging, with an emphasis on reusing packaging that is resistant to change over time. Repurposing glass jars was shown to be a strong consideration for many participants in this study. Lastly, while responses were limited, plastic pouches were associated with trust, convenience, being different, and their larger quantity. Again, it is likely that the visible product damage for the pulses processed within plastic pouches from participant handling resulted in low acceptance. This is an inherent disadvantage of plastic pouches: although lighter and easier to transport (Dlamini et al. 2024), they are more susceptible to damage due to the lack of rigidity and their thin nature. Previous research has indicated consumer awareness of package function and protection, where plastic packaging was viewed as not functional for biscuits due to too many broken biscuits, indicating the package was not protective (Oloyede and Lignou 2021). Again, due to the transparent nature of glass or plastic packaging material, product deformations are more apparent, impacting product quality and allowing for increased consumer inspection (Simmonds et al. 2018).

## 4 Conclusions

It is evident that different packaging materials result in processed pulses with varying levels of quality. Across both pulse varieties, samples that were processed within metal cans had consistently high sensory ratings by participants. Glass jars were viewed to be the highest quality packaging material, identified as being trusted and environmentally friendly and successfully performing necessary package functions while also having satisfactory characteristics. The appreciation for glass by study participants suggests that consumers might be willing to purchase canned pulses in new packaging forms, presenting potential innovation opportunities to generate more interest or higher appreciation for pulses—which are currently undervalued by consumers. The perception of RTE pulses could potentially be elevated through packaging, increasing consumer acceptance of a highly nutritious and sustainable food in premium packaging compared to traditional metal can packaging, incentivizing consumers to choose a more sustainable food group option. In general, consumer idealization of glass packaging as a more sustainable packaging material should be addressed by future research, as these perceptions are not supported by environmental impact data, suggesting a need for increased consumer education on packaging material sustainability to combat generalization. While the results of this study showcased a high affinity for glass packaging, it also demonstrated what pulse quality and packaging attributes consumers value in general—such as a product that has high overall liking, nontransparent packaging, convenience, and packaging that can be resealed. These preferred packaging attributes from this study could be incorporated into future packaging in order to align with consumer expectations and the need for increased packaging sustainability. It is worth noting that the plastic pouches used within this study, while poorly perceived by most participants, could likely be improved. The thin nature of the plastic pouch made the product very susceptible to damage, significantly impacting perceived product quality. For product developers and plant breeders, this information is indicative that pulses should be retort processed within packaging materials with better rigidity and/or shorter processing times to improve product quality, particularly for fast‐cooking yellow bean varieties. Ultimately, to improve the acceptance of RTE retort‐processed pulse products among consumers, product developers should pair pulse varieties with superior sensory attributes and packaging that is sustainable and user‐friendly. Consumer education may be needed either on the packaging itself or through consumer education campaigns to communicate specific sustainability attributes and/or practices.

## Author Contributions

**Lauren Thomas**: conceptualization, investigation, formal analysis, visualization, writing – original draft, data curation. **Nomzamo N. Dlamini**: conceptualization, formal analysis, data curation, writing – review and editing, investigation. **Karen Cichy**: conceptualization, funding acquisition, supervision, writing – review and editing. **Jeffrey Swada**: conceptualization, funding acquisition, supervision, writing–review and editing. **Emily J. Mayhew**: conceptualization, supervision, investigation, writing – review and editing.

## Conflicts of Interest

The authors declare no conflicts of interest.

## Supporting information




**Supplementary Material**: jfds70585‐sup‐0001‐SuppMat.docx
