# Supplementary material for: Toward Sustainable Food and Packaging Choices: Consumer Perception of Quality and Sustainability of Pulses Packaged in Metal, Glass, and Plastic
Source: J Food Sci. 2025 Oct 9;90(10):e70585. doi: 10.1111/1750-3841.70585 (PMC12511835; doi:10.1111/1750-3841.70585)
Supplement: Supplementary file 1 — Supplementary Material: jfds70585‐sup‐0001‐SuppMat.docx [file JFDS-90-0-s001.docx]

**SUPPLEMENTARY MATERIALS**


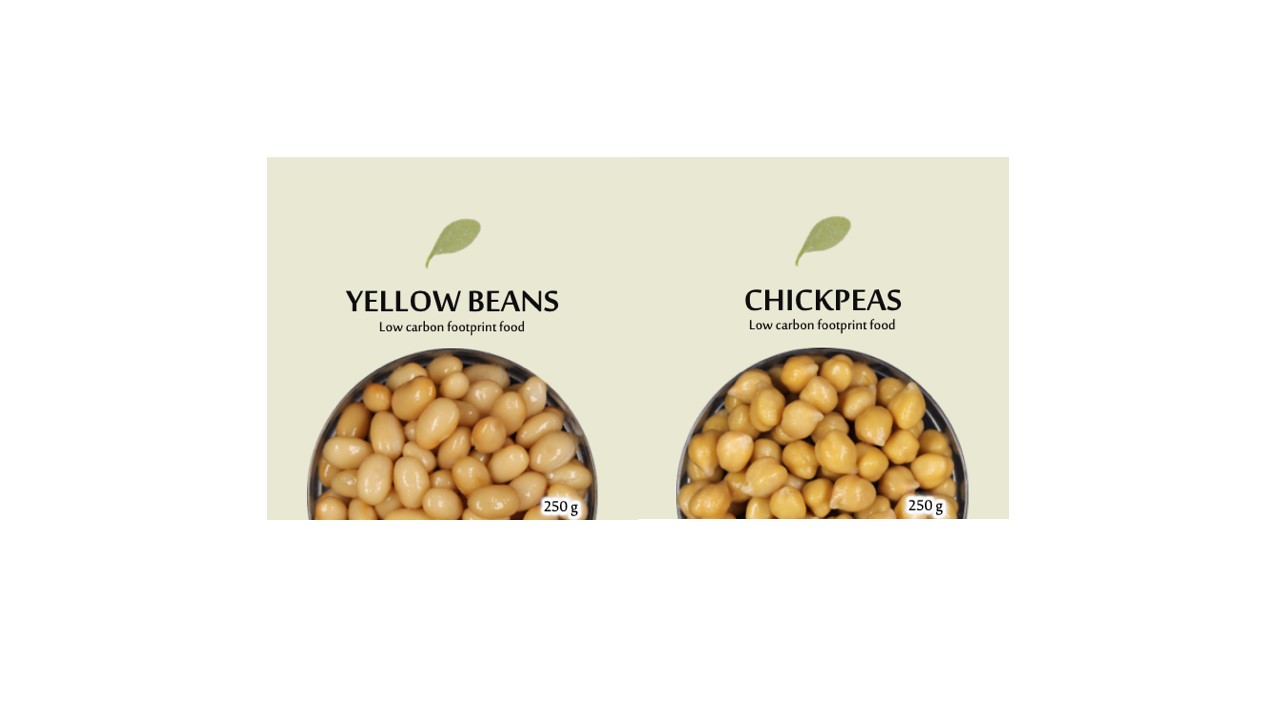


**Figure S1:** Product label placed on metal can, glass jar, and plastic pouches of retort processed yellow beans (left) and chickpeas (right).

**Table S1**: Questions to evaluate participant sustainability attitudes, using a 7-point Hedonic scale, and their corresponding sustainability factors. Question 10 is worded negatively in comparison to the rest of the scale.

| **Question** | **Sustainability Attitude Assessed** |
| --- | --- |
| Q1: Using products with environmentally friendly packaging will reduce environmental pollution and improve the environment | The scale of attitudes toward the purchase behavior of products with environmentally friendly packaging |
| Q2: Using products with environmentally friendly packaging will help reduce the waste of natural resources. |  |
| Q3: Using products with environmentally friendly packaging will help conserve natural resources. |  |
| Q4: I feel comfortable using products with environmentally friendly packaging. |  |
| Q5: My friends think I should use products with environmentally friendly packaging. | The scale of social influence |
| Q6: People important to me encourage me to use products with environmentally friendly packaging. |  |
| Q7: I learned that consuming environmentally friendly packaging products contributes to a better environment. |  |
| Q8: Information in the media encourages me to try products with environmentally friendly packaging. |  |
| Q9: I can protect the environment by buying products with environmentally friendly packaging. | The scale of consumers’ perception of effectiveness |
| Q10: I don’t think I can help solve environmental problems. |  |
| Q11: I think that if I do some environmental protection in my daily life, I will contribute a lot to our environment. |  |
| Q12: I think that if I participate in environmental protection, I will encourage my family and friends to participate too. |  |
| Q13: I am considered obsolete (outdated) if I do not participate in environmental protection. | The scale of personal image concerns |
| Q14: Participating in environmental protection makes me unique among others. |  |
| Q15: I believe that environmentally friendly packaging can protect the product inside. | The scale of quality of environmentally friendly packaging |
| Q16: I think that environmentally friendly packaging can be as beautiful and convey product content as conventional packaging. |  |
| Q17: I think environmentally friendly packaging can attract consumers’ attention as conventional packaging. |  |
| Q18: I believe that environmentally friendly packaging can be as durable as conventional packaging. |  |
| Q19: I want to buy products with environmentally friendly packaging. | The scale of intention to buy products with environmentally friendly packaging |
| Q20: I consider purchasing products with environmentally friendly packaging before making a purchase. |  |
| Q21: I want to encourage people to buy products with environmentally friendly packaging. |  |
| Q22: I want to consume products with environmentally friendly packaging. |  |

**Table S2**: Demographic information of sensory test participant (n = 109).

| **Age** | |
| --- | --- |
| Age (years) | 32.6 ± 16.8 |
| **Gender** | |
| Male | 38% |
| Female | 61% |
| Non-Binary | 1% |
| **Ethnic Background (CATA)** | |
| Asian or Pacific Islander | 28% |
| Native American | 5% |
| Black or African American | 7% |
| Hispanic or Latino | 7% |
| White or Caucasian | 61% |
| Other | 4% |
| **Education Level** | |
| Some High School | 0% |
| High School Graduate or Equivalent | 9% |
| Some College | 32% |
| Trade, Technical or Vocational School | 1% |
| Associate Degree | 4% |
| Bachelor's Degree | 26% |
| Master's Degree | 20% |
| Professional Degree | 2% |
| Doctorate Degree | 6% |
| **Employment Status** | |
| Not Employed | 0% |
| Student | 58% |
| Employed Part-Time | 8% |
| Employed Full-Time | 26% |
| Retired | 8% |
| **Diet Type** | |
| Omnivore | 72% |
| Vegetarian | 7% |
| Ovo-vegetarian | 2% |
| Pescatarian | 2% |
| Flexitarian | 9% |
| Vegan | 1% |
| Other | 6% |
| **Types of Legumes/Pulses Consumed (CATA)** | |
| Chickpeas | 75% |
| Yellow Beans | 38% |
| Navy Beans | 33% |
| Kidney Beans | 75% |
| Pinto Beans | 70% |
| Black Beans | 91% |
| Edamame | 59% |
| Green Beans | 82% |
| Lentils | 72% |
| Other | 9% |
| **Pulse Consumption Frequency** | |
| Most days | 4% |
| 1-3 times per week | 51% |
| 1-3 times per month | 37% |
| 5-10 times per year | 5% |
| Rarely | 4% |
| **Modes of Consumption (CATA)** | |
| Dried | 49% |
| Canned | 86% |
| Frozen | 39% |
| Flour | 14% |
| Paste | 43% |
| Chips | 39% |
| Other | 32% |
